# Magnetic nanocomposite for lead (II) removal from water

**DOI:** 10.1038/s41598-024-68491-8

**Published:** 2024-07-30

**Authors:** Asif Shahzad, Bagher Aslibeiki, Sawssen Slimani, Sagnik Ghosh, Marco Vocciante, Marco Grotti, Antonio Comite, Davide Peddis, Tapati Sarkar

**Affiliations:** 1 Present Address: Department of Materials Science and Engineering, Uppsala University, Box 35, 75103 Uppsala, Sweden; 2 Faculty of Physics, University of Tabriz, Tabriz, Iran; 3 Department of Chemistry and Industrial Chemistry & Genova INSTM RU, University of Genova, 16146 Genova, Italy; 4 Institute of Structure of Matter, National Research Council, nM2-Lab, Via Salaria Km 29.300, Monterotondo Scalo, 00015 Roma, Italy

**Keywords:** Pollution remediation, Magnetic properties and materials, Nanoparticles, Synthesis and processing

## Abstract

A magnetic perovskite-spinel oxide nanocomposite synthesized through a sol–gel self-combustion process is used for the first time as an adsorbent to remove toxic heavy metals (i.e., Pb^2+^). The synthesized LaFeO_3_:CoFe_2_O_4_ ((LFO)_1_:(CFO)_x_) (x = 0.11–0.87) nanocomposites possess good stability, abundant oxygenated active binding sites, and unique structural features, making them suitable for removing divalent Pb^2+^ ions. Scanning electron microscopy, X-ray diffraction, BET surface area, magnetization measurements, zeta-potential analyses, and X-ray photoelectron spectroscopy were used to analyze the nanocomposites, and their structural changes after Pb^2+^ ions adsorption. Batch tests confirmed that (LFO)_1_:(CFO)_x_ efficiently removes Pb^2+^ from water with a maximum adsorption capacity of 105.96 mg/g. The detailed quantitative study indicates that the interaction of hydroxyl groups with Pb^2+^ ions occurs through electrostatic interactions and complex formation. We also demonstrate a new ring-magnetic separator system that allows magnetic separation of the toxic ions at a higher speed compared to traditional block magnets. The unique structure, high porosity, large specific surface area, and oxygenated functional groups of (LFO)_1_:(CFO)_x_ nanocomposites make them promising materials for removal of heavy metal ions and possibly other environmental pollutants. This study provides a new approach to preparing nanocomposites of magnetic spinel ferrites with perovskite oxides for environmental applications.

## Introduction

In today’s world, contamination of freshwater bodies and shortage of clean water are critical issues leading to water security concerns and global unrest^1^. Inorganic pollutants, in particular heavy metal ions in ground and freshwater streams, pose a threat to human health and the environment as they are biologically nondegradable and have a propensity to accumulate in the water environment as well as human body for long periods of time^2^. The rapid population growth, especially in Southeast Asia, and the concomitant rapid industrialization to fulfil the needs of the growing population have led to an enormous increase in freshwater consumption, which in turn, escalates water pollution^3^. Both industrial and domestic uses lead to the release of a wide range of toxic organic/inorganic compounds, including carcinogenic heavy metals into water bodies.

Lead (Pb) is a toxic heavy metal that can cause a range of health problems, including issues with cardiovascular disorders, reproductive problems, kidney damage, high blood pressure, and even an increased risk of high mortality rates as reported by the World Health Organization (WHO)^4^. As a result, it is critical to effectively remove Pb^2+^ ions from Pb-contaminated water/wastewater that is generated by industries such as lead smelting, paint manufacturing, battery manufacturing, mining, and municipal waste incineration^5–7^. Out of the different treatment methods such as chemical precipitation, ion exchange, electrolysis, and adsorption that have been explored for Pb^2+^ removal, adsorption is a highly attractive option due to its higher efficiency, lower cost, simpler operation, ease of regeneration, and selectivity^8,9^. The effectiveness of the adsorption method is due to the desirable physical and chemical characteristics of the adsorbent, including its porous structure, surface area, and availability of binding sites^10^. In the past, numerous adsorbents have been evaluated for their ability to remove Pb^2+^ ions from water but achieving high efficiency and selectivity in the presence of other metal ions has been a longstanding challenge. Although materials like activated carbon^11^, metal–organic frameworks (MOFs)^12,13^, organic polymers, and ion exchange resins^14,15^, have been extensively studied for their ability to act as sorbents for Pb^2+^, major issues such as non-selectivity towards Pb^2+^ ions (soft ions), stability problems, leachability, and low surface area still persist^16,17^. Furthermore, the use of complex compounds such as MOFs and organic ligands, with their long and complex synthesis methods and extensive use of expensive chemicals, are major challenges in commercializing the process.

Perovskite oxides, on the other hand, are characterized by a very stable crystal structure^18^. With the general chemical formula ABO_3_ (where A and B are cations), their distinctive crystal structure is similar to the mineral perovskite (CaTiO_3_). This structure has shown potential for removing heavy metals through adsorption^19^. The unique crystal structure and surface properties of perovskite oxides provide binding sites for heavy metal ions, making them highly attractive for use in water and wastewater treatment. Specifically, perovskite oxides such as LaFeO_3_ and LaMnO_3_, which have a high specific surface area, are effective in removing divalent heavy metals like Pb^2+^, Cu^2+^, and Cd^2+^ from aqueous solutions^19^. The adsorption mechanism involves the formation of metal hydroxide complexes on the surface of the perovskite oxide, resulting in the removal of heavy metal ions from the solution. However, the perovskite nanoparticles (NPs) with particle size in the range of 20–50 nm have shown a great tendency to agglomerate and they are very difficult to separate from water after the adsorption process^20^ that could very easily lead to secondary contamination. To overcome this challenge, in this work, we have synthesized magnetic nanocomposites of a perovskite oxide (LaFeO_3_) with ferrimagnetic cobalt ferrite (CoFe_2_O_4_), showing a method for quick and effortless collection of the adsorbent and toxic metal ions.

To the best of our knowledge, this paper shows for the first time the use of magnetic nanocomposites of LaFeO_3_:CoFe_2_O_4_ ((LFO)_1_:(CFO)_x_) (x = 0.11–0.87) to adsorb harmful heavy metals (e.g., Pb^2+^) from wastewater. Sol–gel self-combustion method^18^ was used to create nanocomposites with varying molar ratios of LaFeO_3_ and CoFe_2_O_4_, and the most effective nanocomposite (keeping in mind both the adsorbent capacity as well as the ease of magnetic separation) was chosen for the removal of the target pollutant. These magnetic nanocomposites have a relatively high surface area and pore volume, and include oxygenated (M–O and M–OH) and surface terminal groups. To investigate the adsorption potential of the materials, a series of experiments were conducted using lead-II (Pb^2+^) as a model toxic metal. An interface interaction mechanism is proposed for the adsorption of Pb^2+^ ions, and the functioning of a lab-scale prototype magnetic separator is demonstrated for magnetic separation of the exhausted adsorbent from water.

## Results and discussion

**Figure 1:**
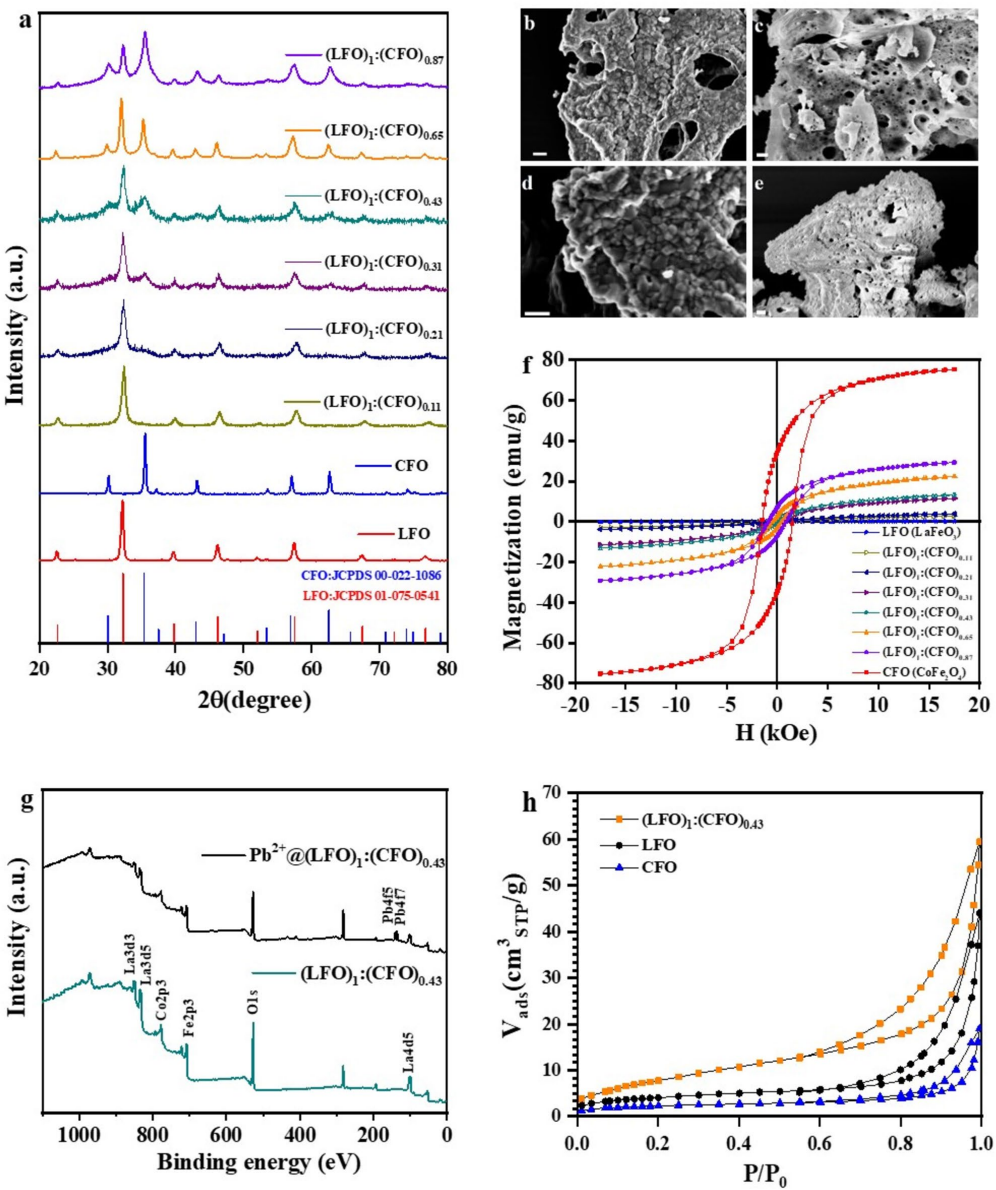
(**a**) XRD patterns of (LFO)_1_:(CFO)_x_ nanocomposites. SEM image (scale bars: 200 nm) of (**b**) CoFe_2_O_4_, (**c**) LaFeO_3_, (**d**) (LFO)_1_:(CFO)_0.43_, and (**e**) Pb^2+^@(LFO)_1_:(CFO)_0.43_. (**f**) Magnetic hysteresis loops of (LFO)_1_:(CFO)_x_ samples recorded at 300 K. (**g**) XPS scan of (LFO)_1_:(CFO)_0.43_, before and after Pb^2+^ adsorption. (**h**) N_2_ adsorption/desorption isotherms of (LFO)_1_:(CFO)_0.43_, LFO, and CFO.

Our previous work reported the efficient and clean synthesis of (LFO)_1_:(CFO)_x_ through sol–gel self-combustion methods^21^, which is significantly faster than hydrothermal treatment and yields similar results. As expected, the physical properties of the (LFO)_1_:(CFO)_x_ nanocomposites were found to vary with changes in the value of x. The XRD patterns of single-phase CoFe_2_O_4_ and LaFeO_3_ showed the formation of spinel (JCPDS card 00-022-1086) and perovskite (JCPDS card 01-075-0541) structures, respectively. In the (LFO)_1_:(CFO)_x_ nanocomposites, all the observed reflections could be indexed to the anticipated phases and were in complete agreement with the LFO and CFO phases (Fig. 1a). All the synthesized nanocomposites, including LFO and CFO NPs, were air-annealed at 500°C in a furnace to achieve well-crystallized phases. The average crystallite sizes estimated by the Scherrer equation (D_XRD_) of the end-phases (LFO and CFO) were ~ 17 and ~ 25 nm, respectively. Based on the XRD investigation, the initial adsorption tests for Pb^2+^ (described in a later section and Table 2), and the magnetization measurements (Fig. 1f), the most promising nanocomposite (LFO)_1_:(CFO)_0.43_ was selected for further exploration (i.e., the sample that presented a reasonable adsorption capacity and a reasonable saturation magnetization value necessary for magnetic separation).

Figure 1b,c show the SEM images of the single-phase end members (CFO and LFO) and reveal a difference in morphology between the two. The SEM images of CFO samples suggest the presence of cuboidal-shaped CoFe_2_O_4_ NPs (Fig. 1b). However, the small NPs of LFO were not visible, which may be attributed to a higher degree of agglomeration (Fig. 1c). Additionally, on the surface of the (LFO)_1_:(CFO)_0.43_ nanocomposite, the LaFeO_3_ phase was found to be covered with CoFe_2_O_4_ NPs, as shown in Fig. 1d. It should be underlined that the SEM images obtained in the study are comparable to previous reports of materials synthesized using similar synthesis methods^22^. The SEM image of the nanocomposite after Pb^2+^ adsorption is also shown in Fig. 1e.

Figure 1f displays the magnetization curves of the nanocomposites recorded at 300 K. CoFe_2_O_4_ shows ferrimagnetic behavior with a large saturation magnetization (M_S_) value of ~ 75 emu/g. Bulk LaFeO_3_ is known to be antiferromagnetic^23^. In the nanocrystalline form, finite size effects can modify the magnetic ground state and lead to weak ferromagnetic behavior, that for small particles can cause superparamagnetism at room temperature^24^. The LFO nanoparticles used in this study show a very low magnetization value (~ 0.184 emu/g), together with complete lack of magnetic saturation (see Fig. S1 in the Supplementary Information), suggesting a dominant antiferromagnetic nature. The presence of a weak ferromagnetic shell in the LFO nanoparticles cannot be discounted, although it will not have any appreciable contribution to the magnetic separation process, the latter being driven completely by the strongly ferrimagnetic CoFe_2_O_4_ component of the nanocomposites. For the different nanocomposites, the magnetization value increased with the increase of CFO phase, as expected (see Supplementary Table S1). After the adsorption process, the sample (Pb^2+^@(LFO)_1_:(CFO)_0.43_) retained its ferrimagnetic character and saturation magnetization value (see Supplementary Fig. S2), thus, ensuring that it could be used for the subsequent magnetic separation process.

X-ray photoelectron spectroscopy (XPS) was utilized to characterize the elemental composition and oxidation states of (LFO)_1_:(CFO)_0.43_. Figure 1g displays the XPS spectra for both pristine (LFO)_1_:(CFO)_0.43_ and Pb^2+^@(LFO)_1_:(CFO)_0.43_, along with their respective atomic concentrations (%), as described in Table 1. The survey spectrum of (LFO)_1_:(CFO)_0.43_ (Fig. 1g) shows the presence of La 3d, Co 2p, Fe 2p, and O 1s. High-resolution spectra of La 3d, Co 2p, and Fe 2p confirm that the La, Co, and Fe atoms possess a formal oxidation valence state of + 3. Further details of the XPS analysis can be found in a later section, where we have described the removal mechanism. The measured atomic percentages of La 3d5, Co 2p3, Fe 2p3, and O 1s were 5.4%, 6.9%, 23.0%, and 64.7%, respectively, and 1.4% of Pb 4f after adsorption in the Pb^2+^@(LFO)_1_:(CFO)_0.43_ sample, which suggests the successful formation of the desired nanocomposite and heavy metal Pb adsorption.

**Table 1:** Atomic concentrations (%) of (LFO)_1_:(CFO)_0.43_ and Pb^2+^@(LFO)_1_:(CFO)_0.43_ measured with XPS analysis.

Adsorbent	Atomic concentration (%)				
	La 3d_5_	Co 2p_3_	Fe 2p_3_	O 1s	Pb 4f.
(LFO)_1_*:*(CFO)_0*.*43_	5.4	6.9	23.0	64.7	–
Pb^2+^@(LFO)_1_*:*(CFO)_0*.*43_	4.9	6.4	20.3	66.9	1.4

The physisorption isotherm of LFO, CFO, and (LFO)_1_:(CFO)_0.43_ shows a hysteresis loop (Fig. 1h) which, following the IUPAC technical report^25^, can be classified as type H3 suggesting a non-rigid aggregate structure with the presence of macroporosity. The (LFO)_1_:(CFO)_0.43_ isotherm is placed higher than that of the LFO and CFO isotherms, indicating a higher specific surface area and its hysteresis shows a larger loop. CFO, LFO, and (LFO)_1_:(CFO)_0.43_ show BET specific surface areas of ~ 8 m^2^/g, ~ 15 m^2^/g, and ~ 30 m^2^/g, respectively. It is noteworthy that the (LFO)_1_:(CFO)_0.43_ sample shows a specific surface area (ssa) twice that of the ssa of single-phase LFO and nearly 4 times that of the ssa of single-phase CFO. This can be ascribed to the unusual synthesis method that we have developed for the nanocomposites. In fact, these materials were synthesized using a simultaneous bi-phasic synthesis technique where both the phases were grown at the same time^18^. This distinct growth process tends to modify the morphology of the two phases, thus, resulting in a higher specific surface area for the nanocomposite compared to the single phases. This synergistic effect is advantageous for the adsorption process and therefore our synthesis method is beneficial for designing nanocomposites for water remediation via adsorption.

### Removal of lead ions

Magnetic nanocomposites with small particle size, high specific surface area, high porosity, and surface hydroxyl groups (i.e., M⎼O and M⎼OH)^26^ could be useful environmental remediation agents, particularly for multivalent heavy metal entrapment for water/wastewater clean-up^27^. To evaluate the Pb^2+^ removal performance, the synthesized nanocomposites were deployed as adsorbents for a Pb^2+^ solution under certain experimental conditions. The Pb^2+^ uptake from the solution was determined for all the (LFO)_1_:(CFO)_x_ nanocomposites through batch adsorption tests. All the adsorbents exhibited significant removal efficiency at lower Pb^2+^ concentrations (20 mg/L), with LFO exhibiting higher removal efficacy as compared to CFO NPs with removal rates of 98.4% and 16.7%, respectively (Table 2). LaFeO_3_ shows a higher tendency to bind divalent Pb^2+^ cations and a similar trend was noticed in the removal efficiency of the other nanocomposites i.e., the adsorption efficiency decreases with increasing amount of CFO in the nanocomposites. This behavior can be probably ascribed to the morphology of the samples. As discussed before, SEM images suggest that the CFO nanoparticles cover the binding sites available on the surface of LFO, resulting in strong magnetic exchange coupling between the two phases^28^ and a decrease in the removal efficiency for samples with higher amount of CFO. Nevertheless, it is worth noting that the nanocomposite with the highest amount of CFO (x = 0.87) still shows a removal efficiency close to 80%.

**Table 2:** Pb^2+^ adsorption by (LFO)_1_:(CFO)_x_ nanocomposites.

Adsorbent	Pb^2+^@(LFO)_1_:(CFO)_x_		
	Initial conc. (mg/L)	Final conc. (mg/L)	Removal (%)
LFO	20.89	0.34	98.4
(LFO)_1_:(CFO)_0.11_	20.89	0.58	97.2
(LFO)_1_:(CFO)_0.21_	20.89	0.67	96.8
(LFO)_1_:(CFO)_0.33_	20.89	2.60	87.6
(LFO)_1_:(CFO)_0.43_	20.89	3.03	85.5
(LFO)_1_:(CFO)_0.65_	20.89	3.64	82.6
(LFO)_1_:(CFO)_0.87_	20.89	4.67	77.6
CFO	20.89	17.41	16.7

**Figure 2:**
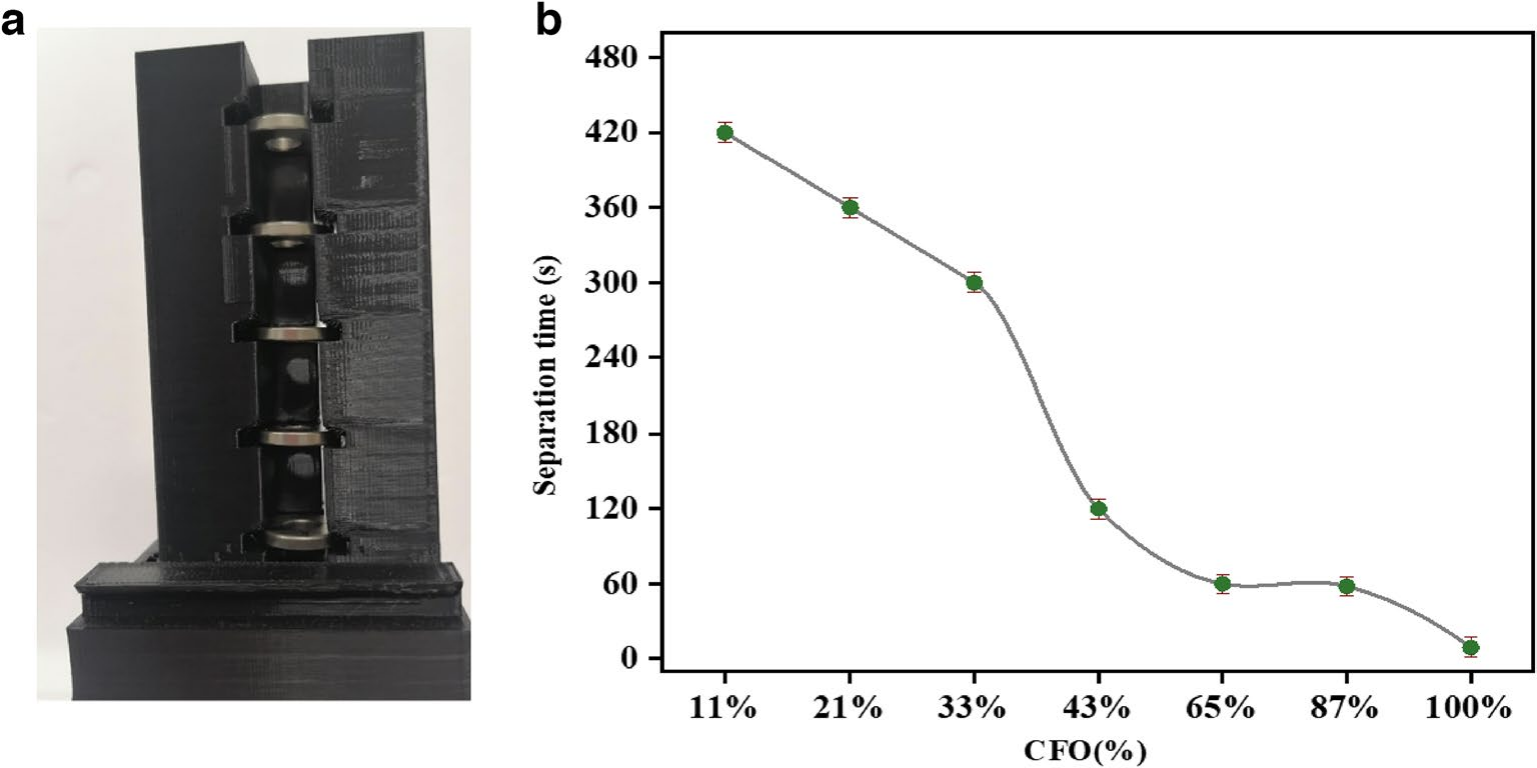
(**a**) Developed ring magnetic separator system. (**b**) Magnetic separation time as a function of the CFO content in the nanocomposites.

On the other hand, the addition of ferrimagnetic CFO allows magnetic separation of the adsorbents after the purification process of the water. A magnetic separator based on commercial ring permanent magnets (NdFeB) has been developed to perform the separation tests in static conditions. The separator (Fig. 2a) is equipped with a series of ring permanent magnets appropriately arranged to produce high field gradient around a glass tube containing the test sample (i.e., water + adsorbent). Simulation with COMSOL software was performed to define the magnetic configuration maximizing the particle separation (see Supplementary Sect. 2). According to Supplementary Eq. (S6), the magnetic force upon a magnetic carrier depends on the spatial gradient of the magnetic field; our simulation optimizes this parameter. According to the identified optimal configuration, the device employed is expected to exert an average value of the radial component of the magnetic field $\overline{\nabla {H }_{r}^{2}}$ = 4.74 × 10^11^ A^2^/m^3^ on the composite materials in the section affected by the rings. From this, the average value of the resulting magnetic force can be estimated given the properties of a particle (more details are provided in the supporting information)^29^. The experiments were performed on samples with different CFO content and for each concentration, the measurements were repeated three times. In a typical experiment, the nanocomposite was mixed with water in a glass tube with a targeted concentration of ~ 6 mg/mL. To guarantee homogeneity, the solution was sonicated for 5 min. Figure 2b shows the separation time dependence of CFO content in the test samples. The materials were separated from the solution (i.e., macroscopic point of view) for all the investigated samples. Nevertheless, the separation time varies by 1–2 orders of magnitude with the CFO percentage, changing from 420 s for (LFO)_1_:(CFO)_0.11_ nanocomposite to 58 s for (LFO)_1_:(CFO)_0.87_ sample and to 9 s for CFO nanoparticles. It should be underlined that the magnetic separation was more rapid using our ring magnetic separator than that using magnetic separators that consist only of one block of permanent magnet (not reported here).

All the tested nanocomposites could be recovered within reasonable time, especially the ones with x ≥ 0.43. Out of these, (LFO)_1_:(CFO)_0.43_ (La:Co = 1:0.43) shows the best adsorption capacity and was, therefore, selected for further adsorption tests, as described below.

**Figure 3:**
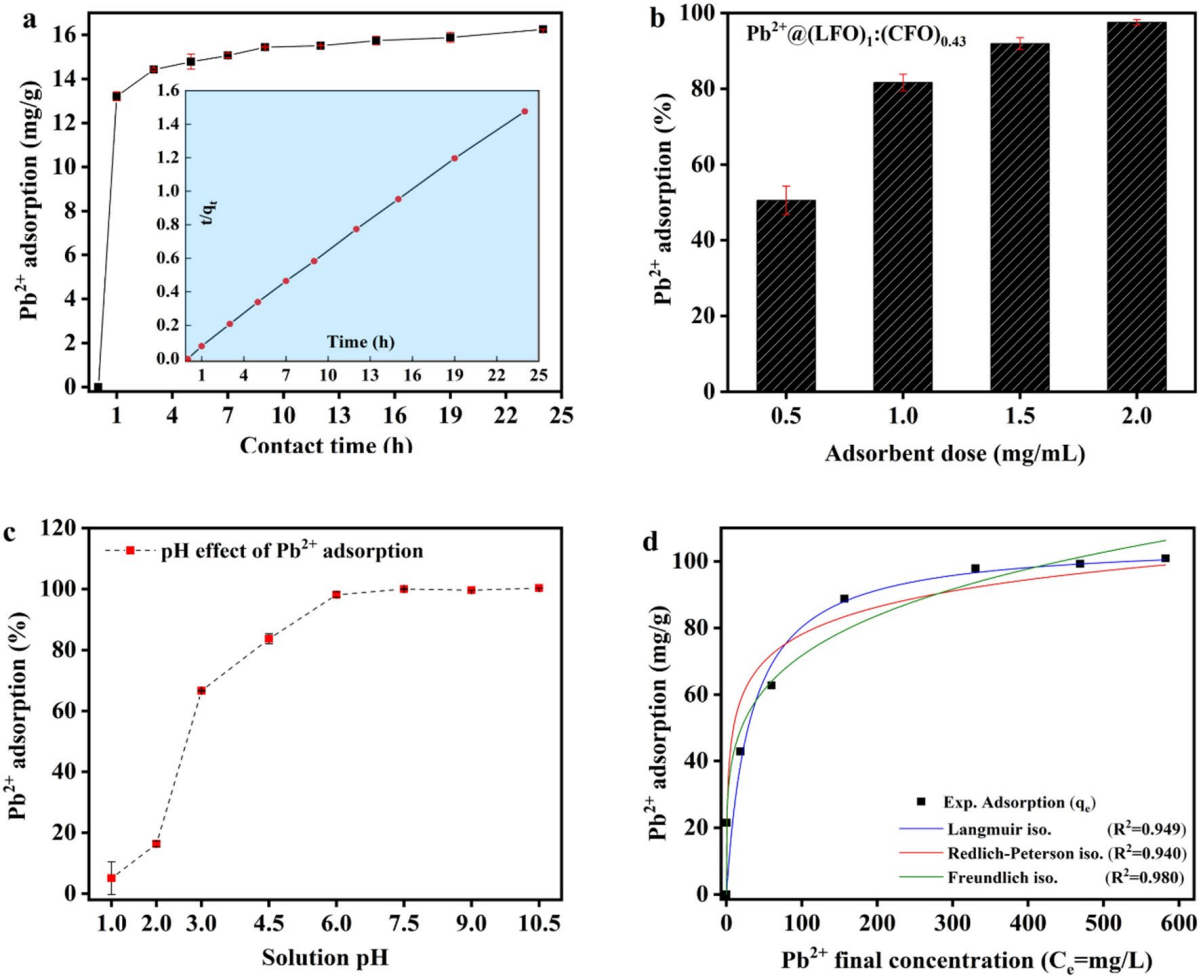
(**a**) Pb^2+^adsorption equilibrium was achieved after 9 h of contact; inset: fitting of the data with the pseudo second-order kinetics model. (**b**) Adsorption efficiency increased with increasing adsorbent dose and at 2.0 mg/mL showed highest removal efficiency. (**c**) The effect of solution pH and (**d**) adsorption isotherm plot for Pb^2+^ adsorption.

The results of the kinetic studies indicate that the adsorption of Pb^2+^ onto (LFO)_1_:(CFO)_0.43_ occurs at a high rate. At a pollutant concentration of 20 mg/L, the reaction equilibrium was established within 1 h, and approximately 66% of the total metal ions were adsorbed within the first hour of the reaction, followed by a slow mass transfer process and achieved equilibrium stage at around 9 h. The rapid adsorption of Pb^2+^ can be attributed to the high surface area, porosity, and densely packed vacant binding sites on LFO. Additionally, the experimental data obtained with various initial Pb^2+^ concentrations were fitted using the pseudo-first-order and second-order kinetics models, as outlined in the Supplementary Sect. 1. The second-order kinetics model provided an excellent fitting of the data (Fig. 3a, inset), and thus describes well the adsorption process of Pb^2+^ onto (LFO)_1_:(CFO)_0.43_, with a higher regression coefficient (R^2^) compared to that obtained using Lagergren first-order kinetic model. The calculated adsorption capacities (q_t_) obtained from the pseudo-second-order kinetics model are in agreement with the experimental maximum adsorption capacities (q_e_). These kinetic observations suggest that the adsorption of Pb^2+^ onto (LFO)_1_:(CFO)_0.43_ is a chemical process with a rate-determining step. Considering the removal efficiency of the adsorption system, the adsorbent dose is another important parameter to consider. For Pb^2+^ adsorption, a higher dose of (LFO)_1_:(CFO)_0.43_ results in a higher removal efficiency, with 1.0 mg/mL resulting in a Pb^2+^ removal efficiency of 80% that increased to 98% when the adsorbent dose was increased to 2.0 mg/mL (Fig. 3b).

The adsorption of heavy metals is generally influenced by the pH level of the solution. To investigate this aspect in the adsorption of Pb^2+^ by (LFO)_1_:(CFO)_0.43_, the adsorption reaction was conducted at different pH values ranging from 1.0 to 10.5. The results showed that only 5.1% and 16.3% of Pb^2+^ were adsorbed at pH 1.0 and 2.0, respectively. However, an abrupt increase in the efficiency of Pb^2+^ adsorption was observed at pH 3.0 (66%), and it reached a maximum adsorption capacity of 98.2% at pH 6. The percentage of Pb^2+^ adsorption remained significantly high (99–100%) in the pH range from 7.5 to 10.5 (Fig. 3c). The effectiveness of adsorption was affected by the form of the Pb^2+^ ions present in solution and the surface properties of the adsorbent. At low pH, the adsorption efficiency of (LFO)_1_:(CFO)_0.43_ was found to be low, which could be attributed to the reduced surface charge on (LFO)_1_:(CFO)_0.43_, leading to a competition between H^+^ and M^2+^ ions for the available surface sites^30^. This may be due to the protonation of the hydroxyl groups on (LFO)_1_:(CFO)_0.43_, which creates repulsive forces in highly acidic media (pH < 3). Furthermore, the zeta potential of (LFO)_1_:(CFO)_0.43_ supports the observed change in adsorption efficiency of Pb^2+^ at different pH values. The point of zero charge (pH_PZC_) is believed to be at pH ⁓ 1.2. (LFO)_1_:(CFO)_0.43_ exhibited a slightly positive zeta charge which changed to higher negative ζ values for pH > 1 (see Supplementary Fig. S5).

Studying adsorption isotherms is a fundamental tool for describing adsorption behavior and determining the adsorption capacity of a material^31^. Adsorption isotherms such as Langmuir, Freundlich etc. are commonly used to study the adsorption of pollutants from water and other fluids^32^. In this study, different isotherm models were used to analyze the experimental dataset of the adsorption of Pb^2+^ onto (LFO)_1_:(CFO)_0.43_. The Langmuir isotherm model suggested a maximum adsorption capacity of 105.96 mg/g (Table 3), which is significantly higher than those reported in literature for spinel ferrites and perovskites such as CoFe_2_O_4_, ZnF_2_O_4_, and NiTiO_3_ (Table 4). This indicates that the synthesized (LFO)_1_:(CFO)_0.43_ nanocomposite is a suitable adsorbent for effectively capturing lead ions from aqueous solutions, with the additional advantage of allowing easy magnetic separation of the adsorbed ions, thereby eliminating secondary contamination. The higher correlation coefficient (R^2^) of the Freundlich isotherm model compared to the Langmuir and Redlich-Peterson isotherm models indicates that the former best describes the experimental data (Fig. 3d). The Freundlich isotherm model assumes multilayer coverage on a heterogeneous surface with multiple adsorption sites for liquid–solid adsorption data^33^.

**Table 3:** Adsorption isotherm parameters for Pb^2+^ adsorption by (LFO)_1_:(CFO)_0.43_ nanocomposite (for the isotherm equations, see supplementary information).

Adsorption isotherm	Parameters	Values	Error	R^2^
Langmuir	K_a_ (L/mg)	0.031	0.0010	0.949
	q_m_ (mg/g)	105.96	6.480	
Freundlich	K_F_ (L/mg)	25.68	3.355	0.980
	1/n	0.0223	2.013	
Redlich-Peterson	A (mg/L)	7.420	5.874	0.940
	B (mg/L)	11.819	1.401

**Table 4:** Comparison of the performance of (LFO)_1_:(CFO)_0.43_ nanocomposite with previous reports of perovskite and spinel ferrites as adsorbent materials.

Adsorbent	Material synthesis route	Adsorption density q_e_ (mg/g)	Crystallite size (nm)	pH	Ref.
CoFe_2_O_4_	Mechanochemical method	20.58	3.8	2	^34^
NiFe_2_O_4_		17.76	19.4		
ZnFe_2_O_4_		9.34	19.2		
LaGdO_3_	Co-precipitation method	392.40	71.0	8	^35^
Porous nano-calcium titanate microspheres (PCTOM)	Citric acid complex sol–gel method	141.8	26.0	6	^36^
Nickel titanate (NiTiO_3_)	Sol–gel method	72	40–50	5	^37^
(LFO)_1_:(CFO)_0.43_	Sol–gel self-combustion	105.4	(11.5):(10.5)	6.5	This study

### Removal mechanism

Nano-sized perovskite oxide and spinel ferrite materials have more reactive sites on their surfaces, which enhances their ability to adsorb pollutants and improves sorption kinetics^38^. The surface of these materials contains hydroxyl groups that are major binding sites for Pb^2+^. The number of hydroxyl groups and their exposure to the target pollutant are affected by the size, morphology, and chemical composition of the nanocomposite, (LFO)_1_:(CFO)_x_, which directly impacts the adsorption efficiency. The aqueous phase adsorption capability of the (LFO)_1_:(CFO)_0.43_ nanocomposite for Pb^2+^ is governed by ion exchange and inner surface complexation phenomena. The adsorption process is influenced by solution pH, as the surface charge changes with varying pH, leading to electrostatic attraction or repulsion between the active sites and Pb^2+^ ions. At pH 3 and above, there is a sudden increase in Pb^2+^ removal efficiency due to increased negative charge on the surface terminal groups.

The adsorption process was affected by the solution pH, near the isoelectric point (pH_PZC_ ⁓ 1.2), the positively charged surface of (LFO)_1_:(CFO)_0.43_ could cause electrostatic repulsion between the active sites and Pb^2+^ ions. At a pH of 1, the composite material exhibited a positive charge with a zeta-potential value of 0.949 (see Supplementary Fig. S5). However, as the pH level increased beyond 1, the surface charge of the composite material became more negatively charged. This change in charge resulted in a sudden and significant increase in the efficiency of Pb^2+^ removal as the pH level rose above 2^39^. The Pb^2+^ removal efficiency at pH 1 and 2 were 5.1% and 16.3%, respectively, but jumped to 66.7% at pH 3 and reached > 98% at pH 6 (see Supplementary Table S2), demonstrating the control of solution pH over the Pb^2+^ adsorption process.

The effect of pH on the removal efficiency can be further understood as follows. In nanoparticles, the surface often has broken bonds of M–O, leading to the formation of active negative (O^−^) or positive (M^+^) sites. Therefore, at the surface, the negatively charged oxygen has high tendency to create chemical bonds with surrounding cations like Pb^2+^ or H^+^. Under acidic environment, the presence of high amounts of H^+^ prevents chemical bonds with Pb^2+^ and therefore the adsorption efficiency is low (as can be seen in Fig. 3c). However, under basic conditions (high values of pH), the presence of OH^−^ (instead of H^+^ ions in the solution) favors the formation of Pb–O bonds, thereby increasing the adsorption efficiency (also seen in Fig. 3c). Furthermore, with increasing number of OH^-^ in the solution (for higher pH values), surface functionalization with more hydroxyl groups on the surface of the composite leads to increased negatively charged binding sites that can adsorb Pb^2+^ ions. Thus, with increasing pH, the surface charge changes due to the presence of different hydroxyl groups, such as –FeOH^2+^ –FeOH to –Fe(OH)^2−^ and even –Fe(OH)_3_^2−^, thereby increasing the possibility of Pb–OH bonding. The presence of Pb–O and Pb–OH bonds has been confirmed in the adsorbent post adsorption from XPS measurements that we discuss in the following.

**Figure 4:**
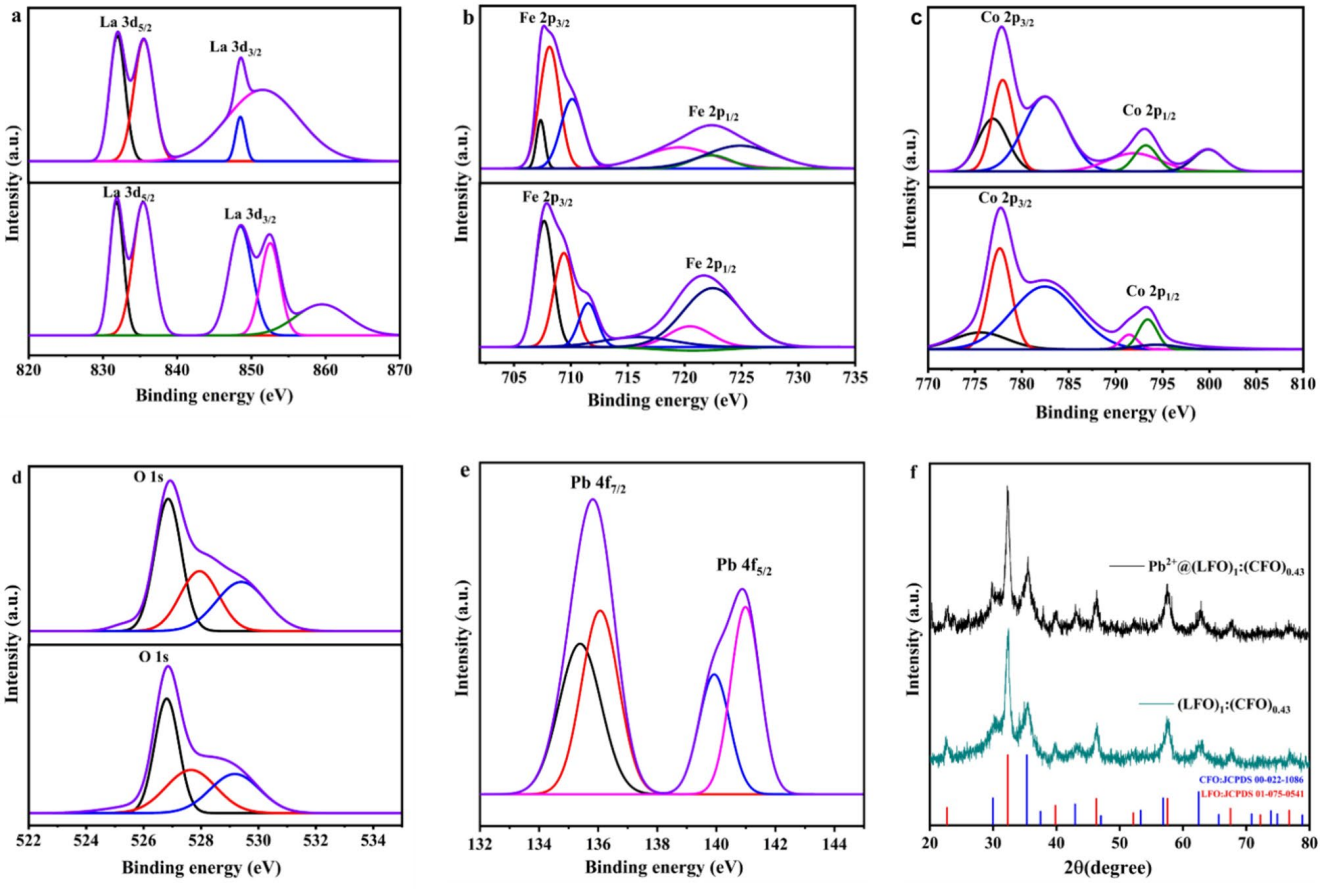
XPS peak fitting analysis of (LFO)_1_:(CFO)_0.43_ (up) and Pb^2+^@(LFO)_1_:(CFO)_0.43_ (bottom) for (**a**) La 3d, (**b**) Fe 2p, (**c**) Co 2p, (**d**) O 1s, and (**e**) Pb 4f. (**f**) XRD patterns of (LFO)_1_:(CFO)_0.43_ before and after Pb^2+^ adsorption.

To shed some light on this phenomenon XPS regional peak fitting analysis on (LFO)_1_:(CFO)_0.43_ before and after adsorption of Pb^2+^ has been performed. The analysis revealed changes in the high resolution spectrum of La 3d, with two strong La peaks at 835.41 and 852.53 eV corresponding to the spin orbital splitting of 3d_5/2_ and 3d_3/2_ of La^3+^ ions in oxide form. After Pb^2+^ adsorption, 3d_5/2_ shifted to a slightly higher binding energy (835.51 eV) while 3d_3/2_ moved to a lower binding energy (851.48 eV), indicating the role of La^3+^ oxide in Pb^2+^ adsorption (Fig. 4a). Similarly the Fe^3+^ oxide peaks observed at 711.53 eV, 720.45 eV, and 724.49 eV belonging to either LaFeO_3_ or CoFe_2_O_4_ in (LFO)_1_:(CFO)_0.43_ nanocomposite (Fig. 4b) corresponds to 2p_3/2_, satellite peak, and 2p_1/2_, respectively. Upon Pb^2+^ adsorption, these peaks shifted to lower binding energies (710.11, 719.57, and 724.85 eV)^40^. Additionally, two Fe^2+^ oxide peaks at 709.38 and 722.49 eV might belong to LaFeO_3_ present in (LFO)_1_:(CFO)_0.43_^40^, which also shifted to lower binding energies (708.12 and 722.32 eV), and a decrease in peak intensity was observed in the post-adsorption spectrum (Fig. 4b). These observations suggest that the oxidation states of Fe^3+^ and Fe^2+^ play a role in Pb^2+^ adsorption.

During Co 2p peak fitting analysis, several peaks were observed including two characteristic peaks at around 777.66 and 782.42 eV which correspond to Co 2p_3/2_ and its shake-up satellites, respectively, as shown in Fig. 4c. The other two peaks at approximately 791.43 and 794.33 eV are Co 2p_1/2_ and its shake-up satellite, respectively, indicate the presence of Co^2+^ ions^41^. Following Pb^2+^ adsorption, the characteristic peaks of Co 2p_3/2_ shifted slightly to higher binding energies (777.96 and 782.47 eV) and Co 2p_1/2_ peaks moved to higher binding energies of 791.98 and 799.88 eV. A more comprehensive understanding of the individual contributions of Co and La in the Pb^2+^ adsorption process requires further investigation, including the calculation of formation energies. Theoretical modeling approaches such as DFT or first-principle studies may provide better insights into these aspects^42^.

The XPS analysis showed a similar trend in peak shifts for O 1s after Pb^2+^ adsorption (Fig. 4d). The peak at 526.80 eV in the O 1s XPS signal is attributed to La–O and Fe–O contributions in the LaFeO_3_/CoFe_2_O_4_ crystal lattice (O_L_), and the relative intensities of the XPS spectra suggest an atomic ratio of 1:1:3 between La, Fe, and O^43^. The O_H_ peak at 529.16 eV is assigned to the hydroxyl group (OH^−^) due to adsorbed moisture from the air. After Pb^2+^ adsorption, both O_L_ and O_H_ peaks shifted to higher binding energies, and a new peak at 529.40 eV could be attributed to the formation of a new bond between Pb^2+^ and the OH group (Pb–O). Although the overall intensity of the O 1s peak increased after the introduction of Pb^2+^, the intensity of the O_L_ peak was higher than that of the O_H_ peak, which may be due to the synthesis method used. In wet-chemical methods such as hydrothermal synthesis, the O_H_ peak intensity is reportedly higher than the O_L_ peak intensity^44^. Therefore, it is assumed that the (LFO)_1_:(CFO)_0.43_ nanocomposites, which were synthesized by a sol–gel method followed by a self-combustion technique, had a lower amount of adsorbed water.

Furthermore, the Pb 4f region displayed doublet two peaks corresponding to 4f_7/2_ and 4f_5/2_ at binding energies of 135.7 eV and 140.0 eV, respectively and with doublet separation of 5.2 eV; these peaks can be assigned to Pb–O or Pb–(OH)_2_ (Fig. 4e). In XPS-elemental analysis, 1.4% atomic concentration of Pb 4f was measured in Pb^2+^@(LFO)_1_:(CFO)_0.43_ (Table 1). The presence of multiple negatively charged functional groups associated with both LaFeO_3_ and CoFe_2_O_4_, and the porosity are the primary factors driving Pb^2+^ adsorption. Guin et al.^45^ reported that a value of 1/n < 1 for the Freundlich constant indicates good adsorption and favorable physical processes. In this study, the values of 1/n and K_F_ were found to be 0.022 and 25.7, respectively. A higher slope for 1/n ranging between 0 and 1 indicates greater adsorption intensity or surface heterogeneity, which demonstrates the synergistic effect of both LaFeO_3_ and CoFe_2_O_4_ in Pb^2+^ adsorption (Table 3). Although perovskite oxides typically exhibit a monolayer adsorption process following the Langmuir isotherm^35^ the presence of CoFe_2_O_4_ in conjunction with LaFeO_3_ appears to create a synergistic effect in Pb^2+^ adsorption. Additionally, XRD analyses confirmed the retention of high crystallinity in (LFO)_1_:(CFO)_0.43_ after Pb^2+^ adsorption. The XRD pattern of Pb^2+^@(LFO)_1_:(CFO)_0.43_ (Fig. 4f) revealed the same crystal phases as those identified in (LFO)_1_:(CFO)_0.43_ prior to adsorption, indicating excellent stability even in an aqueous solution and oxygenated environment, and suitability for multiple cycles after regeneration. In addition, the ferrimagnetic nature of the nanocomposites enables the collection, regeneration, and reuse of the exhausted materials.

## Conclusions

To summarize, we have successfully synthesized ferrimagnetic spinel-perovskite nanocomposites and evaluated their efficiency in removing toxic Pb^2+^ ions. Nanocomposites (LFO)_1_:(CFO)_x_ with different compositions (x = 0.11–0.87) were synthesized, characterized, and tested for their ability to remove Pb^2+^ from water. The value of ‘x’ influenced both the magnetic properties and adsorption efficiency. The (LFO)_1_:(CFO)_0.43_ nanocomposite that exhibited hydroxyl terminal groups, high porosity, and a large surface area were utilized for adsorbing Pb^2+^ ions from aqueous solutions. The adsorption process was pH-dependent, spontaneous, and had a maximum adsorption capacity of 105.96 mg/g. Strong surface complexation and electrostatic attraction contributed to the adsorption of Pb^2+^ onto the (LFO)_1_:(CFO)_0.43_ nanocomposite. The experimental findings suggest that the self-combustion method used to synthesize (LFO)_1_:(CFO)_x_ nanocomposites, which is rapid, low-cost, and effective, may serve as an efficient route towards developing adsorbents for heavy metals and other emerging pollutants. We have also demonstrated the removal of the toxic ions from water post-adsorption using a custom-designed ring-magnetic separator that provides a larger field gradient and hence, is more effective for magnetic separation than single permanent magnets. This illustrates a promising route to tackle the separation problem post adsorption and thereby, eliminate secondary contamination. The easily separable (LFO)_1_:(CFO)_x_ nanocomposites can facilitate large-scale synthesis for the remediation of multiple pollutants, as well as other potential industrial applications.

## Materials and methods

### Materials

Lanthanum(III) nitrate hexahydrate (La(NO_3_)_3_·6H_2_O), iron(III) nitrate nonahydrate (Fe(NO_3_)_3_·9H_2_O), cobalt(II) nitrate hexahydrate (Co(NO_3_)_2_·6H_2_O), nitric acid (69%), and glycine were purchased from Sigma Aldrich. Lead nitrate (99%) was purchased from Alfa Aesar. All reagents were used as received without further purification.

### Synthesis of nanostructures

#### Synthesis of (LFO)_1_:(CFO)_0.43_

We followed a similar synthesis process as described in our previous publication^18^. Two stoichiometric solutions corresponding to LaFeO_3_ (LFO) and CoFe_2_O_4_ (CFO) were prepared separately and then mixed together by adopting the following route: for solution-I, 0.02 mmol of La(NO_3_)_3_·6H_2_O and Fe(NO_3_)_3_·9H_2_O were dissolved in 14 mL deionized water. Next, 1 mL HNO_3_ acid was slowly added to the solution, followed by 0.04 mmol glycine and the solution was stirred for 20 min. Solution-II was prepared by dissolving an appropriate amount of Co(NO_3_)_2_·6H_2_O and Fe(NO_3_)_3_·9H_2_O (Co:Fe = 0.02:0.04 mmol) in water. Next, 1 mL of HNO_3_ was added slowly, followed by 0.006 mmol glycine. In the next step, solution-I and solution-II were mixed and stirred for 20 min at room temperature. The temperature was then increased to 80 °C and maintained for 20 min. Subsequently, the temperature of the hot plate was increased to 150 °C to achieve gel formation. Finally, for the self-combustion reaction, the temperature was increased to 250 °C. The black coloured flakes obtained after the self-combustion process were collected, ground to powder, and subjected to annealing in air at 500 °C for 10 h in a furnace (ramp rate = 15 °C/min). The magnetic nanocomposite obtained after the annealing process was stored in borosilicate vials for further use. Magnetic nanocomposites with different phase fractions i.e., different values of x, (LFO)_1_:(CFO)_x_, were fabricated by changing the concentration of Co against La. The same synthesis procedure was used for the different samples.

### Pb^2+^ adsorption experiments

To evaluate the heavy metal removal capabilities of the synthesized nanocomposites, Pb^2+^ solution was prepared by dissolving a certain amount of lead nitrate in deionized water and the removal performance was assessed via typical batch adsorption testing under appropriate conditions.

#### Adsorbent comparison test

For this test, 10 mL Pb^2+^ (20 mg/L) solution was taken in a 50 mL centrifuge tube and 10 mg (LFO)_1_:(CFO)_x_ adsorbent was added to the solution and agitated for 24 h at room temperature. Afterward, aliquots were drawn and subjected to inductively coupled plasma optical emission spectroscopy (ICP-OES) analysis to determine the remaining concertation of Pb^2+^ in the solution. For the ICP-OES analysis, all samples were filtered through a syringe filter (hydrophilic polyvinylidene fluoride, PVDF, 0.22 µm) and acidified with 2% HNO_3_. The adsorption capacity of Pb^2+^ at equilibrium (q_e_) and the percentage of Pb^2+^ removal (R(%)) were calculated using Eqs. (1) and (2), respectively.

1 $${q}_{e}=\frac{\left({C}_{0}-{C}_{e}\right)\times V}{m},$$

2 $$R(\%)=\frac{\left({C}_{o}-{C}_{e}\right)}{{C}_{o}}\times 100,$$

where, *C*_*o*_ and *C*_*e*_ (mg/L) are the initial and final concentrations of Pb^2+^, respectively, *V* is the solution volume (L), and *m* is the mass of the adsorbent (g).

#### Adsorption kinetics test

For the adsorption equilibrium study, 30 mL of Pb^2+^ (20 mg/L) was mixed with 30 mg (LFO)_1_:(CFO)_0.43_ nanocomposite and agitated for 24 h at room temperature. Aliquots were drawn at different time intervals, filtered, and analyzed via ICP-OES.

#### Effect of solution pH

To examine the effects of pH, the experiments were conducted in a pH range of 2.0–10.5; 0.1 M NaOH or HCl was used to adjust the pH of the solution, while the solution concentration, volume, and adsorbent amount were kept fixed. The zeta-potential analysis of the selected adsorbents LFO, CFO, and (LFO)_1_:(CFO)_0.43_, were performed at different pH solutions.

#### Effect of Pb^2+^ concentration

To identify the point of saturation, experiments were conducted using Pb^2+^ solution concentrations in the range of 20–684 mg/L, while the other influencing parameters were kept constant. The collected aliquots were subjected to appropriate dilution with 2% HNO_3_ prior to ICP-OES analysis.

### Structural, morphological, and magnetic characterization

X-ray powder diffraction (XRD) patterns of the nanocomposites were obtained using an X-ray diffractometer (XRD, D8CuK_α1_, operating at 40 kV and 200 mA over a scanning range of 20°–80°). The XRD patterns were used to check for phase purity and also to estimate the average crystallite size of the samples, using the Scherrer equation^46^.

The morphology of the samples was studied using a field emission scanning electron microscope (SEM–Zeiss LEO 1530 with Oxford AZtec EDS system). To analyze the surface elemental composition of the pristine nanocomposite and metal ion laden samples, scanning X-ray micrographs by Kratos AXIS Supra + X-ray photoelectron spectrometer (XPS, monochromated X-ray sources, Al Kα (1486.6 eV), Ag Lα (2984.3 eV) were collected. A vibrating sample magnetometer (LakeShore 7404 VSM) was utilized to perform magnetic measurements. The measurements included recording room temperature magnetic hysteresis loops within the magnetic field range of ± 18 kOe.

DLS measurements were performed using a Malvern Zetasizer Nano ZS equipped with 633 nm red laser and operating at an angle of 90°. The software used to collect and analyze the data was Zetasizer software version 7.2 from Malvern. 5 mg of each sample was dissolved in 5 mL of deionized water and mixed for 24 h. 1 mL of the supernatant of each sample was then measured using polystyrene cuvette. The measurements were made at a controlled temperature of 25 °C; for each sample, 11 runs of 10 s were performed, with 3 repetitions. The intensity size distribution, the Z-average diameter (Z-ave) and polydispersity index (PDI) were obtained from the autocorrelation function.

Physisorption with N_2_ at 77 K and the evaluation of the BET specific surface area were carried out by an ASAP2020 MP Plus (Micromeritics, USA). The samples were outgassed at 180 °C for 4 h prior analysis.

### Supplementary Information

Supplementary Information.

## Data Availability

The datasets used and/or analysed during the current study are available from the corresponding authors on request.
