# Supplementary material for: Magnetic nanocomposite for lead (II) removal from water
Source: Sci Rep. 2024 Jul 30;14:17674. doi: 10.1038/s41598-024-68491-8 (PMC11291739; doi:10.1038/s41598-024-68491-8)
Supplement: Supplementary file 1 — Supplementary Information. [file 41598_2024_68491_MOESM1_ESM.pdf]

Supplementary Materials

Magnetic nanocomposite for lead (II) removal from water

Asif Shahzad[1], Bagher Aslibeiki[2,1], Sawssen Slimani[3,4], Sagnik Ghosh[1], Marco Vocciante[3], Marco Grotti[3], Antonio Comite[3], Davide Peddis[3,4]* and Tapati Sarkar[1]*

[1]Department of Materials Science and Engineering, Uppsala University, Box 35, SE-75103 Uppsala, Sweden

[2]Faculty of Physics, University of Tabriz, Tabriz, Iran

[3]Department of Chemistry and Industrial Chemistry & Genova INSTM RU, University of Genova, 16146 Genova, Italy

[4]Institute of Structure of Matter, National Research Council, nM2-Lab, Via Salaria km 29.300, Monterotondo Scalo 00015, Roma, Italy

*Corresponding author

Tel.: +46 18 471 3359

E-mail: tapati.sarkar@angstrom.uu.se; davide.peddis@unige.it

1. Isotherm models for lead(II) adsorption on $(LFO)_1$:$(CFO)_{0.43}$

Langmuir isotherm equation[1]:

$$q_e = \frac{q_m K_a C_e}{1 + K_a C_e} \quad \text{(S1)}$$

Freundlich isotherm equation[2]:

$$q_e = K_F C_e^{(\frac{1}{n})} \quad \text{(S2)}$$

Redlich-Peteraon isotherm equation[3]:

$$q_e = \frac{A C_e}{1 + B C_e^g} \quad \text{(S3)}$$

where, q_e is the sorption capacity of $(LFO)_1$:$(CFO)_{0.43}$ at equilibrium, C_e is the equilibrium concentration of Pb^{2+} in solution, q_m is the maximum adsorption capacity, and K_a, K_F, n, A, B, and g are the isotherm constants for the Langmuir, Freundlich, and Redlich-Peterson isotherm model equations.

Kinetics models for Pb^{2+} adsorption on $(LFO)_1$:$(CFO)_{0.43}$

$$\ln(q_e - q_t) = \ln q_e - k_1 t \quad \text{(S4)}$$

where, q_t is the metal ion concentrations at time t and k_1 is the first-order rate constant (s^{-1}).

$$\frac{t}{q_t} = \frac{1}{k_2 q_e^2} + \frac{t}{q_e} \quad \text{(S5)}$$

where, k_2 represents the second-order rate constant (g/mg/h); in this case, t/q is plotted against t. The equilibrium rate constants of the linearized Lagergren pseudo first- and second-order kinetic models were expressed by plotting time t (h) against $\ln(q_e - q_t)$ and t/q_t, respectively.

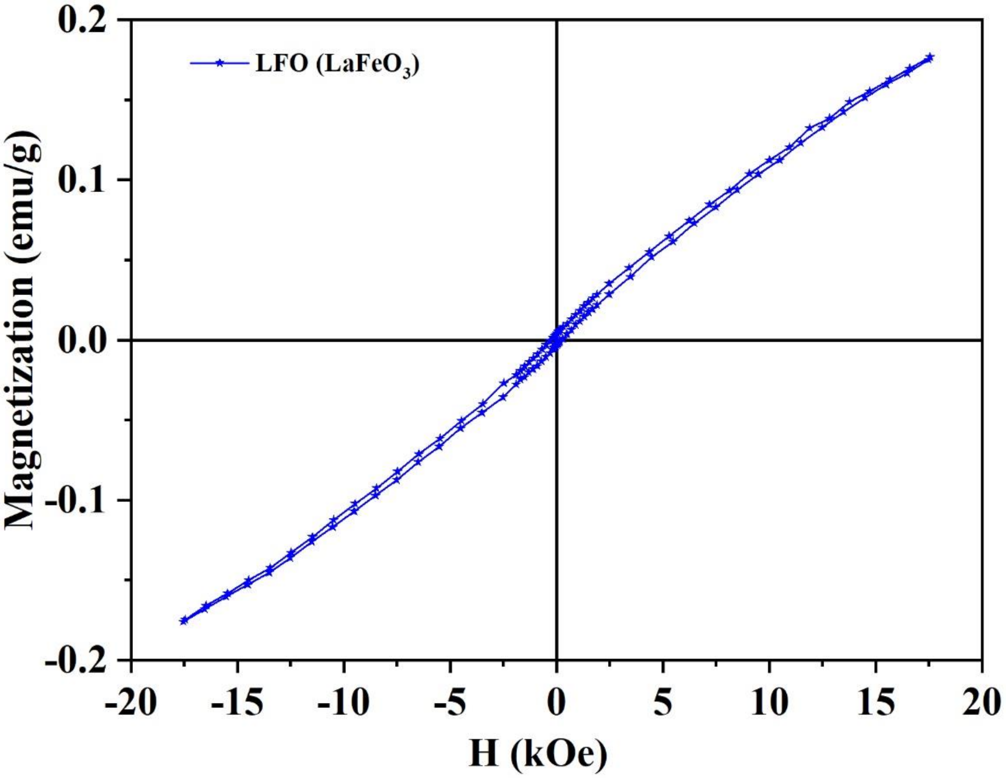

Figure S1. Field dependence of magnetization of $LaFeO_3$ at T = 300 K.

Table S1: La:Co ratios and magnetization at H = 18 kOe for the different nanocomposite samples.

<table>
<tr><th>Sample</th><th>Concentration of Co (%) against La (%) detected from ICP-OES analysis</th><th rowspan="2">Magnetization (emu/g)
at H = 18 kOe</th></tr>
<tr><td colspan="2">For ICP-OES analysis, a certain amount of nanocomposites was added into 2 mL of 70% HNO_3 and stirred for 48 h to ensure digestion. The solution was further diluted 45-times with DW, filtered using syringe filter (0.2µm), and subjected to ICP-OES analysis for Co and La detection.</td></tr>
<tr><td>LFO</td><td>—</td><td>0.18</td></tr>
<tr><td>$(LFO)_1:(CFO)_{0.11}$</td><td>10.63</td><td>2.86</td></tr>
<tr><td>$(LFO)_1:(CFO)_{0.21}$</td><td>21.04</td><td>3.83</td></tr>
<tr><td>$(LFO)_1:(CFO)_{0.33}$</td><td>33.04</td><td>11.50</td></tr>
<tr><td>$(LFO)_1:(CFO)_{0.43}$</td><td>43.38</td><td>13.15</td></tr>
<tr><td>$Pb^{2+}@(LFO)_1:(CFO)_{0.43}$</td><td>43.38</td><td>14.66</td></tr>
<tr><td>$(LFO)_1:(CFO)_{0.65}$</td><td>64.90</td><td>22.30</td></tr>
<tr><td>$(LFO)_1:(CFO)_{0.87}$</td><td>87.30</td><td>29.30</td></tr>
<tr><td>CFO</td><td>—</td><td>75.28</td></tr>
</table>

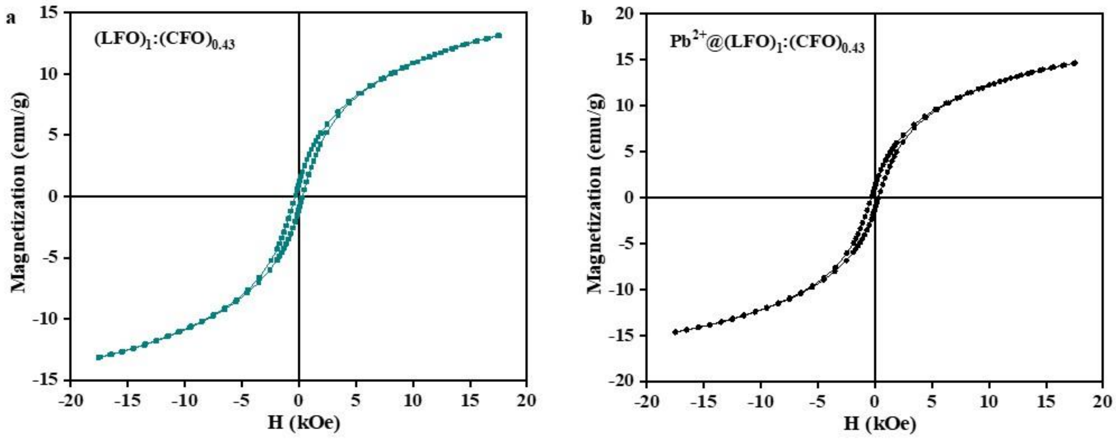

Figure S2. Magnetization hysteresis loop of $(LFO)_1:(CFO)_{0.43}$ (a) before and (b) after Pb^{2+} adsorption.

2. Computer-aided preliminary device design

Computational simulations were carried out using COMSOL Multiphysics software in order to design a prototype device for the magnetic separation of nanocomposites using an external magnetic field.

NdFeB ring permanent magnets with features based on commercial products (grade N48) were considered. In order to maximize the resulting overall magnetic force, which will be responsible for the magnetic separation of the material within the liquid, in the simulations a variable number of such NdFeB magnetic rings were arranged around a section of a glass tube.

Specifically, two setups were considered: 3 and 5 rings. For each of the two cases, a parametric study was conducted by varying the mutual distance of these rings in the range of 1/4 to 4 times the ring thickness (i.e., mutual distance from 2 to 32 mm). The magnetic polarization of the rings (always parallel to the glass tube axis) has been considered both concordant or alternate.

For each of the two cases, the overall induced magnetic force was evaluated while varying polarization and ring spacing. This value was calculated as the integral in the relevant channel section (blue part in Fig. S3) of the H-field square gradient radial component. This value represents the radial component of the net magnetic force, dependent on the nanocomposite characteristics, such as the particle volume V_{NP} and its magnetic susceptibility χ_{NP} (equation S6)[4].

$$F_M = V_{NP} \cdot \chi_{NP} \cdot \nabla H^2 \rightarrow \frac{F_M}{V_{NP} \cdot \chi_{NP}} = \nabla H^2 \quad \text{(S6)}$$

Figure S3 shows a sketch of one of the two setups considered in the simulations.

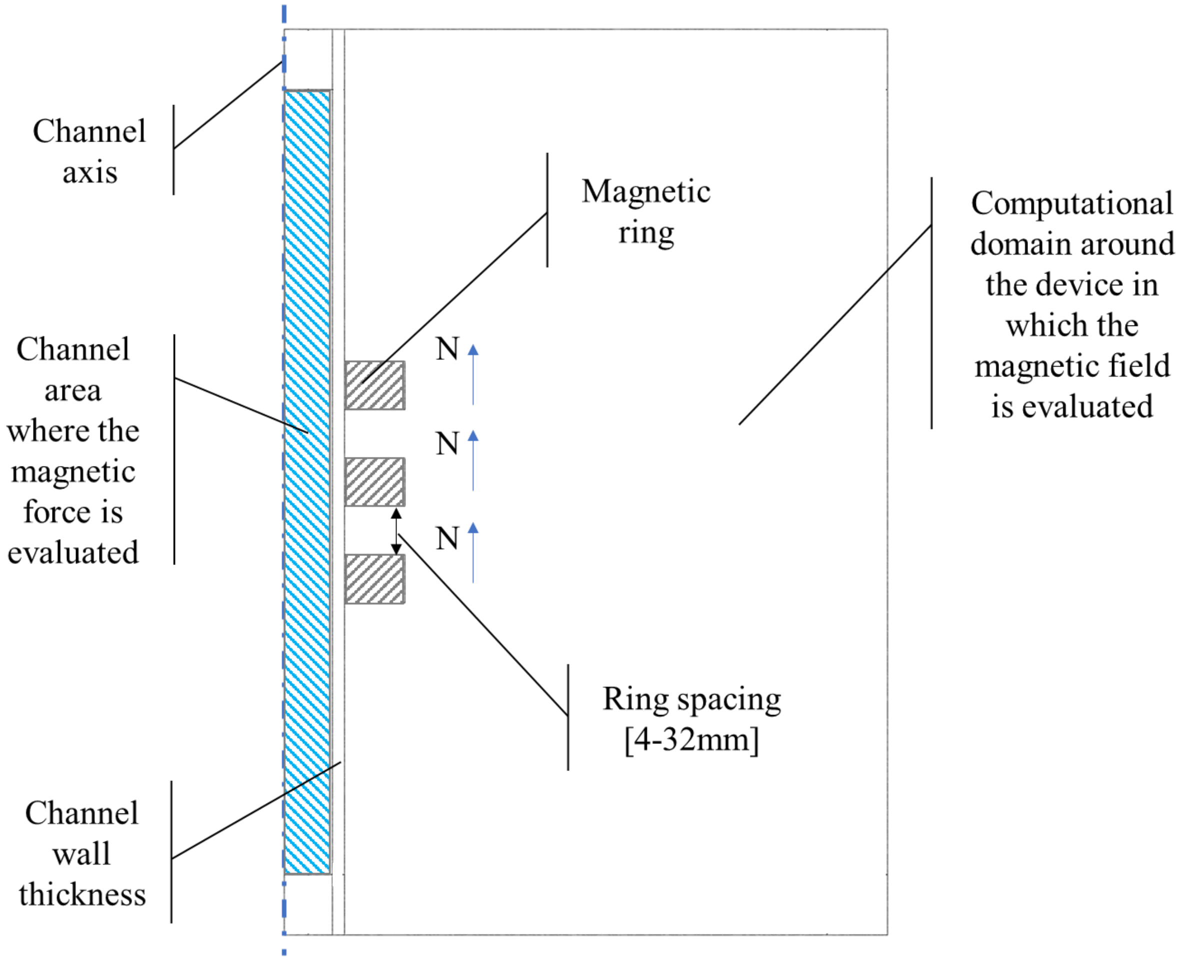

Figure S3. Axisymmetric 2D representation of the computational domain for the 3-ring system.

Figure S4 shows the integral of the H-field square gradient radial component (i.e., the net radial component of the magnetic force induced by the magnets) for the 3- and 5-ring system as the distance between the rings varies. Only the case of concordant polarization between the rings is reported, being always better than the alternate case. Data are reported in terms of total value (Fig. S4a) and normalized value (Fig. S4b). The normalization is intended with respect to the contribution to the magnetic force generated by a single ring, i.e., the ratio of the actual total value to the "theoretical" total value, understood as the product of the value of the single ring times the number of rings in the system.

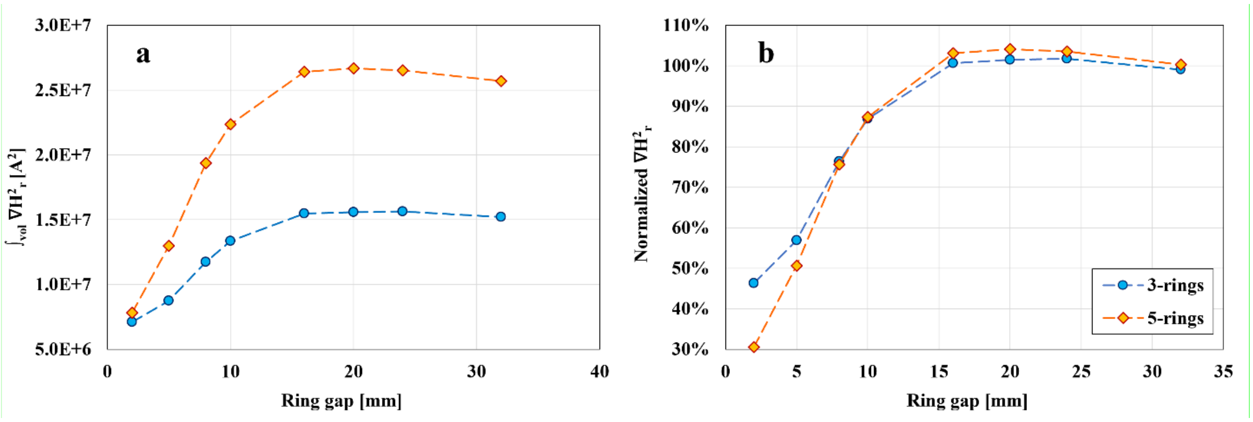

Figure S4. (a) Volumetric integral of the radial component of ∇H_r^2 determining the total magnetic force F_M felt by the nanocomposites in the glass tube section affected by the rings; (b) percentage value of ∇H_r^2 normalized with respect to the theoretical value generated by a single ring.

Interestingly, the obtained results demonstrate that the magnetic fields generated by the single rings interact either destructively or constructively as a function of the distance between them (Fig. S4b). Moreover, there is an optimal distance at which the synergistic effect given by the constructive interaction is maximized. This distance was found to be 24 and 20 mm for the 3- and 5-ring configuration, resulting in a gain of approximately 2% and 4%, respectively, compared to the simple additive contribution of the fields (Fig. S4b). For any given configuration, knowing the volume of the glass tube section affected by the rings, it is possible to derive the average value $\overline{\nabla H_r^2}$, which, in the optimal case of the 5-ring configuration is equal to 4.74×10^{11} A^2/m^3. Given the size of a particle and its magnetic susceptibility, the average value of the magnetic force exerted on the particle (which changes at each point in the domain) remains defined by the above equation S6.

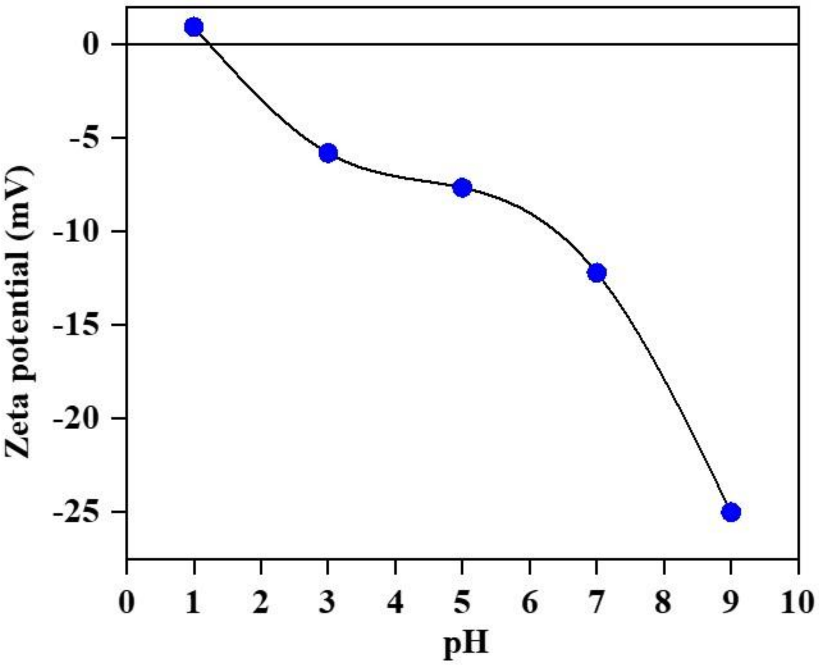

Figure S5. Zeta-potential values of $(LFO)_1$:$(CFO)_{0.43}$ in DI water at different solution pH.

Table S2. Effect of solution pH on Pb^{2+} adsorption on $(LFO)_1$:$(CFO)_{0.43}$ nanocomposite.

**Solution pH**	**Pb^{2+}@$(LFO)_1$:$(CFO)_{0.43}$**				
	Initial Conc. (mg/L)	***Final Conc. (mg/L)***	***Standard deviation***	***Adsorption capacity (mg/g)***	***Removal (%)***
1	18.06	17.14	0.96	0.92	5.1
2	22.36	18.71	0.19	3.65	16.3
3	23.16	7.72	0.05	15.44	66.7
4.5	22.86	3.73	0.38	19.13	83.7
6	21.89	0.40	0.20	21.49	98.2
7.5	21.78	0.00	0.05	21.78	100
9	21.89	0.08	0.00	21.81	99.6
10.5	18.73	0.00	0.00	18.79	100

References

1. Langmuir, I. The constitution and fundamental properties of solids and liquids. Part I. Solids. *Journal of the American chemical society* **38**, 2221-2295 (1916).
2. Freundlich, H. Over the adsorption in solution. *J. Phys. chem* **57**, 1100-1107 (1906).
3. Redlich, O. & Peterson, D. L. A useful adsorption isotherm. *Journal of physical chemistry* **63**, 1024-1024 (1959).
4. Panina, L. V. *et al.* Spatial Manipulation of Particles and Cells at Micro-and Nanoscale via Magnetic Forces. *Cells* **11**, 950 (2022).
